# The analgesic effect of electroacupuncture on acute thermal pain perception-a central neural correlate study with fMRI

**DOI:** 10.1186/1744-8069-7-45

**Published:** 2011-06-07

**Authors:** Shivshil Shukla, Artour Torossian, Jeng-Ren Duann, Albert Leung

**Affiliations:** 1 Anesthesia Service, Veteran Administrations San Diego Healthcare System; 3350 La Jolla Village Drive, MC 125, San Diego, CA92161, USA; 2 Vanderbilt University, School of Medicine; D-3300 Medical Center North, Nashville, TN 37232-2104, USA; 3 Swartz Center for Computational Neuroscience, Institute for Neural Computation, The University of California, San Diego; 9500 Gilman Drive # 0559, La Jolla, CA 92093-0559, USA; 4 Department of Anesthesiology, The University of California, San Diego, School of Medicine; Veteran Administrations San Diego Healthcare System; 9300 Campus Point Drive, A-113, MC 7651, La Jolla, CA 92037-1300, USA

## Abstract

**Background:**

Electrical acupuncture (EA) has been utilized in acute pain management. However, the neuronal mechanisms that lead to the analgesic effect are still not well defined. The current study assessed the intensity [optimal EA (OI-EA) vs. minimal EA (MI-EA)] effect of non-noxious EA on supraspinal regions related to noxious heat pain (HP) stimulation utilizing an EA treatment protocol for acute pain and functional magnetic resonance imaging (fMRI) with correlation in behavioral changes. Subjects underwent five fMRI scanning paradigms: one with heat pain (HP), two with OI-EA and MI-EA, and two with OI-EA and HP, and MI-EA and HP.

**Results:**

While HP resulted in activations (excitatory effect) in supraspinal areas known for pain processing and perception, EA paradigms primarily resulted in deactivations (suppressive effect) in most of these corresponding areas. In addition, OI-EA resulted in a more robust supraspinal sedative effect in comparison to MI-EA. As a result, OI-EA is more effective than MI-EA in suppressing the excitatory effect of HP in supraspinal areas related to both pain processing and perception.

**Conclusion:**

Intensities of EA plays an important role in modulating central pain perception.

## Background

Recent studies which explored the potential use of acupuncture in preemptive and acute pain analgesia indicated that acupuncture could reduce intraoperative opioid requirement and improve postoperative pain control [1-4]. However, the neuronal events leading to the analgesic effect of acupuncture on acute pain perception are largely unknown. In the context of acute pain treatment, a particular acupuncture treatment system called the tendinomuscular meridian has been described in acupuncture textbooks as an effective means to treat acute pain located in extremities [5-7]. A previous study adopting this acute pain acupuncture treatment paradigm and a thermal human experimental pain model with peripheral sensory testing have demonstrated that a short duration of unilateral electrical acupuncture (EA) at one lower extremity could transiently reduce heat pain (HP) perception with corresponding thermal threshold elevations in bilateral lower extremities, suggesting this acupuncture treatment protocol may have a direct effect on central neuromodulation of pain [8]. A follow-up study using the same treatment protocol with an extended duration of stimulation resulted in a prolonged period of analgesic benefit [9]. However, aside from the stimulation duration effect, how the intensity of the stimulation may affect the analgesic and corresponding central neuromodulatory effect is still unknown. Clinically, the adequate dosing of acupuncture is commonly assessed in terms of the intensity of deqi sensation. Therefore, it is mechanistically important to understand how acupuncture may affect cortical neuromodulation in the acute thermal pain state based on the intensity of the stimulation. Here we hypothesize that the neuromodulatory effect of acupuncture on acute pain is stimulation intensity dependent and different intensities of stimulation will result in different degrees/patterns of cortical activation which subsequently result in different degrees of alteration in pain perception when stimulated with the same intensity of noxious thermal simulation. To test our hypothesis, we conducted the current study utilizing a published acute pain treatment acupuncture paradigm, thermal noxious stimulation and functional magnetic resonance imaging (fMRI), to: 1) assess the baseline supraspinal blood oxygen level dependent (BOLD) response related to HP stimulation and two (minimal and optimal) intensities of EA with behavioral correlation in pain and deqi (tingling sensation) VAS scores respectively; and 2) assess the direct effect of the different intensities of EA on supraspinal response to subsequent HP stimulation. The authors are aware of different sensations associated with deqi sensation and chose "tingling sensation" as the primary acupuncture intensity assessment based on a previous study which established this particular sensation as the most prominent sensation associated with EA [10].

## Results

10 right-handed normal subjects (6 females and 4 males) were enrolled for the study.

### Thermal threshold and HP VAS scores

The average threshold (°C ± SD) for cold, warm, cold pain and heat pain for the subjects (n = 10) were 27.8 ± 1.8, 35.9 ± 2.1, 11.2 ± 10.2 and 46.5 ± 1.1 respectively. The average HP VAS scores (± SD) in the HP only, HP with minimal intensity EA (MI-EA) and HP with optimal intensity EA (OI-EA) paradigms were 41.0 ± 8.0, 37.5 ± 8.9 and 13.8 ± 4.6 respectively. Paired samples T-test showed significant (P < 0.05) differences in HP VAS scores between HP only and HP with OI-EA paradigms, and also between HP with MI-EA and HP with OI-EA paradigms. However no statistically significant difference was observed between HP only and HP after MI-EA VAS scores.

### EA intensities and Deqi sensation

For the stimulation intensity assessment, the average deqi VAS scores (± SD) for MI-EA and OI-EA were 13.1 ± 8.1 and 59.9 ± 8.8 respectively. For the EA scanning paradigms, the deqi VAS scores were 12.5 ± 7.8 and 61 ± 8.6 for the MI-EA and OI-EA respectively. The average numerical intensities for MI-EA and OI-EA were 3.0 ± 0.5 and 8.0 ± 0.5 respectively on a 0-10 dialing scale. The difference between the MI-EA and OI-EA deqi VAS scores was statistically significant (P < 0.001).

### fMRI findings

**Figure 1:**
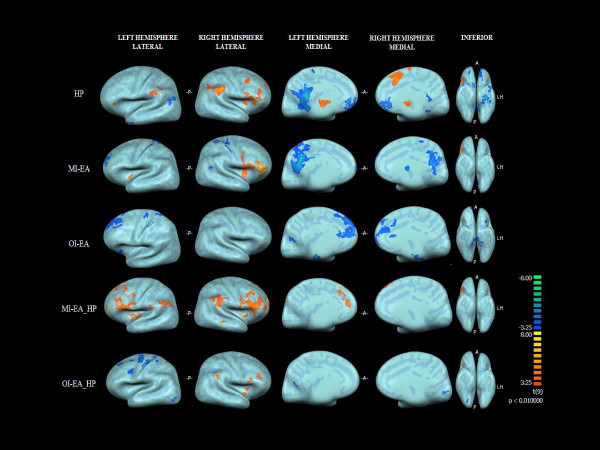
**The inflated group (n = 10) cortical representation of brain activations and deactivations (P < 0.01) in all five paradigms**. HP: heat pain; MI-EA: Minimal intensity electrical acupuncture; OI-EA: Optimal intensity electrical acupuncture; A: Anterior; P: Posterior; LH: Left Hemisphere.

An inflated cortical representation of identified brain areas of activation (positively correlated BOLD signal) and deactivation (negatively correlated BOLD signals) in all five paradigms are shown in Figure 1. The result of within-group and between-group analyses is summarized as follows:

### HP only

The within-group random effects analysis of the HP only paradigms (Table 1) showed significant (P < 0.01) activations at the right thalamus, right secondary somatosensory cortex (SII) and right parietal lobe (BA7,39), right insular cortex (BA13), right premotor cortex(BA6&44), bilateral frontal association cortex (BA9), right dorsal anterior cingulate cortex(BA 32) and left parietal lobe (BA39), and deactivations at the left medial frontal cortex(BA11), left temporal limbic cortex (BA38), left lateral temporal lobe(BA21) and left occipital lobe (BA19).

**Table 1:** Brain regions of activities related to Heat Pain (Rt: Right; Lt: Left; SII: Secondary Somatosensory cortex)

Regions of activities (Peak t-value)	Brodmann Area (cluster size)	Peak Coordinates x, y, z
**Activation**		

Rt. SII, Insular Cortex, Parietal Cortex (7.14)	7, 13, 39 (9350)	47,-47,33

Rt. Lateral Premotor Cortex (7.67)	44 (3875)	44,1,12

Rt. Premotor Cortex (5.41)	6 (2048)	38,13,39

Rt. Frontal Association Cortex (4.26)	9 (446)	26,49,30

Rt. Thalamus (7.23)	N/A (8643)	-22,-17,6

Right Anterior Cingulate Cortex (6.51)	32 (3424)	5,16,36

**Deactivation**		

Lt. Frontal Association Cortex (-4.70)	9 (360)	-43,43,36

Lt. Parietal Lobe (-6.11)	39 (3677)	-64,-50,21

Lt. Medial Frontal Cortex (-11.56)	11 (4267)	2,52,-6

Lt. Temporal Lobe (-4.86)	38 (649)	-46,13,-30

Lt. Lateral Temporal Lobe (-6.53)	21 (425)	-64,-2,-9

Lt. Occipital Lobe (-4.65)	19 (390)	-49,-80,12

### Electrical acupuncture (OI-EA and MI-EA)

The within-group random effects analysis of the MI-EA paradigm (Table 2) showed significant (P < 0.01) activations at the right caudate nucleus, right dorsolateral prefrontal cortex (BA46) and right inferior frontal cortex (BA44) and the left frontal association cortex (BA45), and significant (P < 0.01) deactivation at the right SI (BA1), bilateral SII (BA5 &7), bilateral frontal cortices (BA10), and bilateral dorsal posterior cingulate cortex (BA31). On the other hand, the within-group random effects analysis of the OI-EA paradigm showed only significant (P < 0.01) deactivations at the right SII (BA7), left dorsal anterior cingulate cortex (BA32), left premotor cortex(BA6&8), left Raphe nucleus, left parietal lobe (BA39) and bilateral amygdale.

**Table 2:** Brain Region of activities related to Minimal(MI-EA) and Optimal(OI-EA) intensities of EA (Rt: right; Lt: Left; SI: Primary somatosensory cortex; SII: Secondary Somatosensory cortex)

EA Intensity Mode	Regions of Activities (Peak t-value)	BA (cluster size)	Peak coordinate x, y, z
	**Activation**		

MI-EA	Rt. Inferior frontal cortex (14.92)	44(3453)	47,10,1

	Rt. Dorsal lateral prefrontal cortex(10.71)	46(3233)	45,13,21

	Rt. Caudate Nucleus(6.40)	(270)	17,3,0

	Lt. inferior frontal lobe(6.98)	44 (1514)	-31,22,12

	**Deactivation**		

MI-EA	Rt. SI (-5.15)	1(324)	29,-35,48

	Rt. SII (-5.37)	5(374)	17,-42,51

	Rt. Thalamus (-5.02)	(627)	4,-3,10

	Rt. cingulate cortex (-4.12)	31(15465)	-13,-47,33

	Rt. Frontal lobe(-4.57)	9(591)	11,55,42

	Lt. Frontal lobe(-8.13)	31(8778)	-7,-70,21

	Rt.SII(-4.81)	7(520)	14,-58,36

	Rt. Frontal lobe(-4.37)	10(272)	5,67,27

	Lt. Frontal lobe(-4.76)	10(325)	-4,67,9

	Lt. SII(-4.59)	7(277)	-7,-63,48

	Lt. Frontal lobe(-5.10)	10(540)	-10,56,37

	**Activation**		

OI-EA	**No significant finding**		

	

	**Deactivation**		

OI-EA	Rt. SII(-4.70)	7(273)	38,-68,57

	Lt. Anterior cingulate cortex(-4.20)	32(565)	-10,28,17

	Lt. Premotor cortex(-4.74)	8(2240)	-13,31,45

	Lt. Premotor cortex (-5.45)	6(420)	-25, 13,57

	Lt. Raphe nucleus(-5.77)	(1156)	-1,-20,-15

	Lt. Parietal lobe(-4.41)	39 (374)	-55,-47,48

	Rt. Amygdala (-5.512)	(248)	-68,-75,15

	Lt. Amygdala (-4.91)	(173)	-43,-11,-36

**Figure 2:**
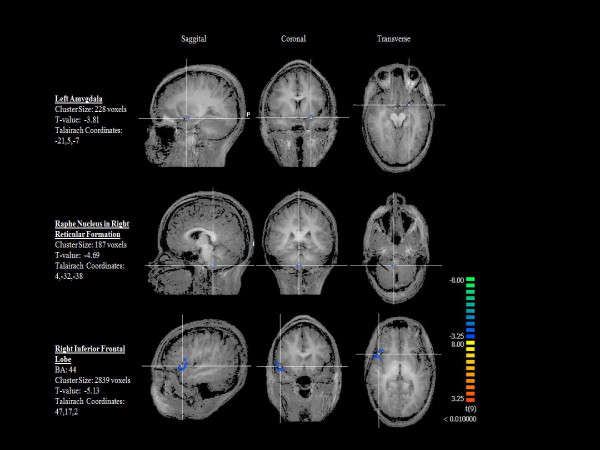
**Brain regions with significant (P < 0.01) deactivation with OI-EA > MI-EA group comparison**.

Subsequently, the between-group random effects analysis (OI-EA > MI-EA) showed OI-EA achieved a significant (p < 0.01) degree of deactivation than MI-EA (Figure 2) in the left amygdala, right Raphe nucleus within the reticular formation, bilateral frontal lobes (BA6, 8, 10 11) and right inferior frontal lobe (BA 44).

### Heat pain with electrical acupuncture

**Figure 3:**
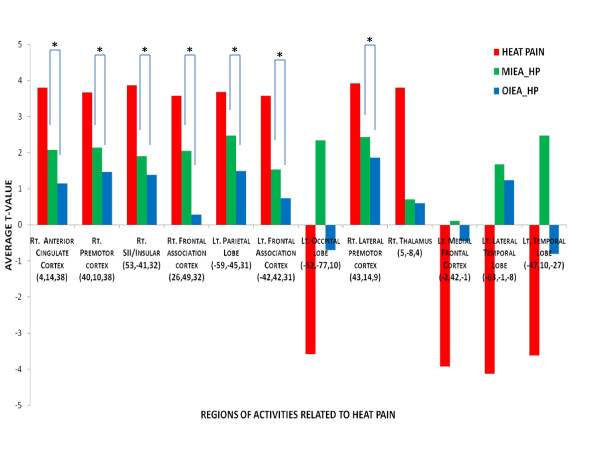
**Comparison of the average T-value in the heat pain (HP) related regions (average voxel x, y, z coordinates) after the two different modes of EA: minimal intensity electrical acupuncture (MI-EA) and optimal intensity electrical acupuncture (OI-EA)**. * indicates regions with significant difference in EA × Intensity interaction between the two paradigms.

**Figure 4:**
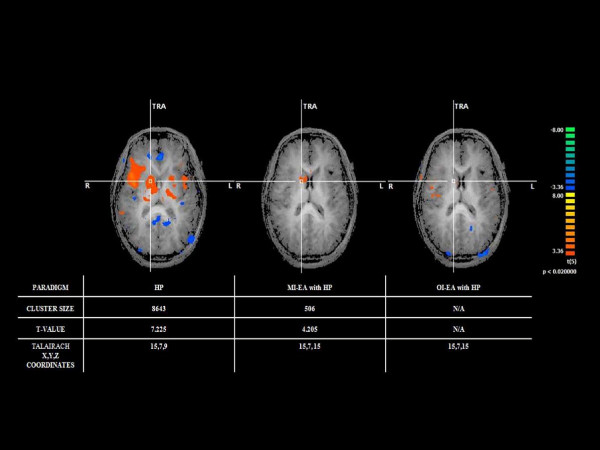
**The relative degree of right thalamic activation observed with heat pain (HP), HP with minimal intensity electrical acupuncture (MI-EA) and HP with optimal intensity electrical acupuncture (OI-EA)**.

In the HP with MI-EA within-group random effects analysis (Table 3), significant activations were found (P < 0.01) at the right thalamus, right caudate nucleus, right frontal lobe(BA47), right prefrontal association cortex(BA10), right limbic association cortex (BA29), bilateral visual cortices (19) and left dorsal lateral prefrontal cortex. No significant areas of deactivation were extracted from the analysis. In the HP with OI-EA within-group random effects analysis, significant (P < 0.01) activations were extracted at the right premotor cortex (BA44), right parietal association cortex (BA39) and right visual association cortex (BA37). For areas of deactivation, the analysis showed a significant effect (P < 0.01) at the left primary somatosensory cortex (SI/BA3), left SII (BA7), and left premotor cortex (BA6 &8). To assess both the overall treatment and the intensity effect of EA on HP, a second level 2-factor (EA &intensity) ANOVA analysis was then performed to assess the specific areas of activation identified with HP stimulation for the two HP with EA paradigms. Except for bilateral frontal association cortex (BA 39), all the other areas including SII, insular, right premotor cortex, anterior cingulate cortex showed a significant (< 0.01) EA × intensity effect. The average t-values of these heat pain related brain regions after the two different EA paradigms were shown in Figure 3. In addition, to assess the effect of EA on the spinothalamic pain pathway, a side-by-side comparison(Figure 4) was also conducted to assess the effect of MI-EA and OI-EA on the thalamic activation induced by HP and showed that OI-EA completely suppressed the HP-induced thalamic activation, whereas, MI-EA moderately reduced the thalamic activation.

**Table 3:** Brain regions of activities related to Heat Pain given under Minimal and Optimal intensities of EA(MI-EA: minimal intensity electrical acupuncture; OI-EA: optimal intensity electrical acupuncture; Rt: Right; Lt: Left)

EA Intensity	Regions of Activities (Peak t-value)	BA (cluster size)	Peak coordinate (x, y, z)
	**Activation**		

MI-EA	Lt. Dorsal lateral prefrontal cortex (3.908)	46(618)	-33,58,21

	Rt. Caudate Nucleus (2.003)	(647)	8, 6, 15

	Rt. Prefrontal Cortex (2.211)	10 (381)	28,49,27

	Rt. Frontal lobe (4.868)	47(298)	41,31,-9

	Rt. Visual Cortex (5.618)	19 (515)	50,-62,6

	Lt. Visual Cortex (3.096)	19 (2090)	-57,-59,27

	Rt. Limbic Area (1.459)	29(506)	5,-26,-9



	**Activation**		

OI-EA	Rt. Premotor (8.225)	44(4703)	47,10,-3

	Rt. Parietal Cortex (3.144)	39(921)	56,-44,24

	Rt. Occipital Cortex (2.194)	37(559)	56,-44,0

	**Deactivation**		

OI-EA	Lt. Secondary Visual Cortex (-2.289)	18(682)	26,-95,-12

	Lt. Secondary Visual Cortex (-3.498)	18(736)	-4,-77,24

	Lt. Premotor Cortex (-1.228)	8(595)	-19,25,33

	Lt. Premotor Cortex (-1.776)	6(421)	-22,7,39

	Lt. SI (-1.699)	3 (416)	-28,-17,48

	Lt. SII (-1.645)	7(334)	-37,-38,33

## Discussion

Recent studies with human experimental pain and functional imaging techniques have provided insightful information regarding areas of the central nervous systems that may play a role in encoding noxious stimulation [11-15]. These pain-related regions-of-activities (ROA) include SI and SII, thalamus, insular cortex, cerebellum, amygdale, prefrontal cortex, premotor and motor cortex. It is also known that collateral pain ascending pathways exist and individual afferent neurons often project in more than one of these pathways [16-18]. These pathways include a spinohypothalamic pathway, a spinopontoamygdaloid pathway, and a component of the spinothalamic pathway that projects to specific midline thalamic nuclei which further projects to limbic cortical areas such as the anterior cingulate cortex (ACC) and insular cortex (IC). Another component of the spinothalamic pathway projects to somatosensory relay nuclei of the thalamus that further relays nociceptive information to SI and SII, which are anatomically interconnected with a ventrally directed cortico-limbic somatosensory pathway that integrates somatosensory input with other sensory modalities such as vision and audition, and learning and memory. In addition, from SI and SII the pathway projects into the posterior parietal cortical areas and the insular cortex, and from the insular cortex to amygdala, perirhinal cortex (medial temporal lobe, BA35 &36), and hippocampus [19]. The latter supraspinal components also have a dual convergence from the ascending spinopontoamygdaloid pathway. These pain related ROA can be further functionally divided into the lateral and the medial systems [20-24]. The lateral system, which is thought to be responsible for the initial noxious signal encoding, carries a somatosensory/discriminatory sensory function, and consists of SI, SII, premotor cortex, motor cortex, and cerebellum, whereas, the medial system, composed of the amygdala, cingulate gyrus and insular cortex, is thought to underlie the affective component of the pain experience. In addition, other cortical areas located in the prefrontal cortical region are thought to play a role in pain modulation [25-28]. In the current study, the areas of supraspinal activities detected from HP stimuli delivered via the block imaging design is consistent with the previous studies.

In the area of acupuncture research, functional imaging studies with various settings of non-noxious acupuncture demonstrated activities in the somatosensory components of pain and deactivations in the affective areas of pain processing such as amygdala, hippocampus and cingulate cortex [29-32]. Although these previous functional imaging studies have distinguished the difference in supraspinal activities between non-painful and painful deqi sensations, how the intensity of non-noxious EA may affect subsequent supraspinal activities in HP modulation is unknown. To the authors' best knowledge, this is the first fMRI study to assess the preemptive effect of acupuncture on central acute thermal pain processing. In the current study, HP alone predominantly induced supraspinal response in the somatosensory (thalamus, contralateral SII), emotional (ACC, insular) and modulatory (bilateral prefrontal cortices, contralateral premotor cortex) components of pain perception. These findings are consistent with results from previous functional imaging studies as discussed above.

In the two EA paradigms with different intensities, significant differences in the supraspinal response between the OI-EA and MI-EA paradigms were noted. First, OI-EA appeared to induce a predominantly deactivation response at the ipsilateral cortical regions that were related to HP processing, whereas, MI-EA induced a mixture of activations and deactivations in both ipsilateral and contralateral hemispheres. Second, while both EA paradigms resulted in a similar degree of deactivation in the SII and ACC, the result of the between-group comparison (OI-EA > MI-EA) demonstrated that the OI-EA generated a much more significant level of deactivation in the contralateral Raphe nucleus, inferior frontal cortex and amygdala, suggesting a more effective role of OI-EA in suppressing the limbic (medial pain pathway) system in comparison to the MI-EA. Moreover, in the HP with EA paradigms, HP with OI-EA resulted in deactivations of the ipsilateral somatosensory cortex(SI, SII) and insular cortex, and no activation in the medial limbic system, whereas, HP with MI-EA resulted in activations in the right limbic system, left dorsal lateral prefrontal cortex, right caudate nucleus, right prefrontal and frontal cortex. In addition to the observed medial pain pathway suppression, this contrast in analgesic effect between MI-EA and OI-EA can be further attributed by the observation that OI-EA can more effectively suppress HP induced contralateral spinothalamic activation as shown in Figure 4. Therefore the overall result from the current study suggests that OI-EA preemptively induces a general sedative effect in supraspinal areas that are essential for HP perception and this effect does not appear to be mediated via any known supraspinal pain modulatory components as none of the prefrontal cortical areas have been activated under OI-EA. In addition, this intensity dependent sedative/suppressing effect is equally effective in both the medial and the lateral pain pathways as the ANOVA showed a significant EA × Intensity interaction effect in several HP related supraspinal areas including the ACC, right SII, insular cortex and premotor cortex.

Previous studies demonstrated that the neuronal analgesic mechanisms of acupuncture were primarily mediated via A-delta afferent fibers which were known to modulate C-fiber mediated HP transmission [33-37]. However, the precise level of neuronal interaction is largely unknown. The result of the current study suggests that EA if given at the optimal non-noxious intensity induces a general supraspinal suppression state which in turn counteracts or minimizes the subsequent excitatory state caused by HP induced C-fiber mediated activation via the spinothalamic and spinopontoamygdaloid pain pathways. This current observation can be added to the overall understanding in the central neuromodulatory mechanisms of EA mediated analgesia.

## Conclusion

In short, by adopting an acupuncture treatment paradigm for acute thermal pain and an experimental HP paradigm, we demonstrated the intensity dependent analgesic effect of EA and the corresponding supraspinal activities. EA at optimal non-noxious intensity can generate a general sedative state in supraspinal areas related to both affective and discriminatory aspects of pain perception and this preemptive supraspinal response is essential in preventing the excitatory state associated with subsequent noxious stimuli.

## Methods

With the Institution Human Subject Protection Committee approval, healthy volunteers were enrolled for the studies based on the following inclusion and exclusion criteria:

Inclusion criteria:

Age 18 to 80;

Male and female;

No analgesics for the past 2 weeks;

Absence of neuropathic pain states;

Exclusion criteria:

History of psychological illness;

History of claustrophobia;

Lack of ability to understand the experimental protocol and to adequately communicate in English;

Pregnancy;

Pending litigation;

History of head trauma, history of trauma or surgery to lower extremities or low back;

History of any metallic implant in the body as listed in the Institute fMRI Center screening list;

### Pre-scanning neurosensory threshold assessment

To be consistent in the study, the location of the thermal thresholds measurement and stimulation was marked at the medial aspect of the left calf between the 6^th ^and 7^th ^marking of an elastic band which consisted of a total of 13 increments, extending from the medial malleolus to the medial tibial plateau. Non-noxious and noxious thermal thresholds including cold and warm, cold and hot pain thresholds were measured by using a Thermal Sensory Analyzer (Medoc Advanced Medical Systems, Minneapolis). This device consisted of a thermode measuring 46 × 29 mm. The temperature of the thermode could either rise or fall (at a rate of 1.2 degrees Celsius/sec for cold and warm sensations, and 3 degrees Celsius/sec for cold and hot pain), depending on the sensations that were being tested. The subject signaled the onset of feeling the tested sensation by pressing a switch, which in turn reversed the temperature change and returned the temperature of the thermode to the 32 degree Celsius baseline. The computer then recorded the temperature of the thermode when the switch was pressed. The average value of testing result was automatically calculated by the computer and displayed on the screen. This method of peripheral sensory testing has been well established in literature and has been used extensively in pain-related studies [8,9,38-40].

### FMRI scanning

**Figure 5:**
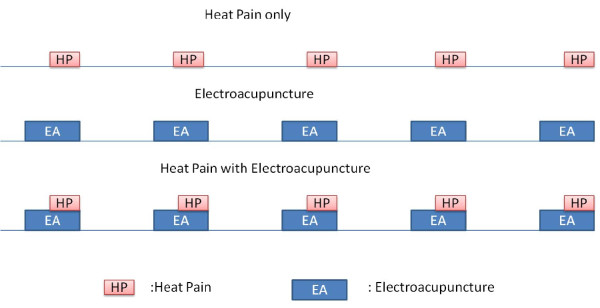
**The study paradigms with heat pain (HP), two intensities of electrical acupuncture (MI-EA &OI-EA) with and without HP**.

Subjects were placed comfortably in a supine position in a scanner with their eyes covered by an eye shield. A Facial-cervical Collar Restraint (FCCR) Device was applied to minimize head movement [41]. The following 5 scanning paradigms (Figure 5) were conducted in a random order:

1) HP stimuli with pre-measured subject-specific HP thresholds were delivered to the subjects' premarked locations at the left medial calves for 15 seconds with a 60-second of baseline temperature (32°C). The stimulus was repeated four times in a box-cart fashion. The heat pain was provided in an oscillating pattern at the target heat pain threshold ± 0.5°C per oscillation with a total of 5 oscillations per stimulation block to avoid pain wind-up via a fMRI-compatible thermode. At the end of the scanning session, the subjects were asked to rate the overall heat pain score on a VAS score.

2) For the two EA stimulation paradigms, one-inch-long, 36G fMRI-compatible gold plated sterile needles were inserted at the LR1 and SP1 points. The location and method of needle placement used in the study were described in the previously published studies [8,9]. Both the needles and grounding electrodes were linked to a 6-volt ES-160 (Electro-Therapeutic Devices Inc., Markham, Ontario, Canada) clinical acupuncture stimulation device, which consisted of a digital display of the stimulation paradigm. Electrical stimulation was provided at a constant frequency of 5 Hz with a pulse width of 300 microseconds. A stimulation intensity assessment session was conducted prior to the scanning. For the MI-EA stimulation threshold determination, the amplitude of the stimulation was gradually increased from 0 to the level when the subject first noticed the tingling sensation. The process was repeated twice. The average stimulation intensity in a 0-10 range and the patients' average VAS rating of deqi sensation (degree of tingling) were recorded. For the optimal intensity EA (OI-EA) stimulation threshold determination, the amplitude was increased from 0 to a maximally tolerated intensity without any sharp pain or discomfort.

After the sensation of EA has completely subsided, the scanning sessions were then conducted. Two separate scanning runs were conducted in a random order for the two different intensities. EA was given for 15 seconds with 60 seconds of resting period in between stimulations. The stimulation was repeated 4 times to complete the scanning session. At the end of each scanning session, the subjects were asked to rate the intensity of tingling sensation felt during the study.

3) Two separate runs of 15 seconds of EA with two different intensities followed by 15 seconds of HP stimulation at subjects' specific heat pain thresholds and 60 seconds of baseline temperature at 32°C were conducted with 4 repetitions to complete the scanning sequence. The subjects were asked to rate the intensity of overall HP at the end of scanning sessions.

In between each scanning paradigm, a minimal of 15 minutes of washout period was provided to ensure either the HP sensation or the EA related deqi sensation had completely subsided.

FMRI Images were obtained via a 3T GE scanner with T2*- weighted EPI-sequence (TE = 30 ms, TR = 2.0 s, α = 90°, TH = 4 mm, 32 slices, FOV = 220 × 220 mm^2^, MA = 64 × 64). Two T1-weighted images were acquired: one for spatially normalizing the functional images and the other for anatomical details.

### Behavioral data analysis

A paired sample *t*-test was used to compare the VAS scores of hot pain and *de qi *sensations.

### fMRI data analysis

Each individual subject's functional and anatomical data sets were processed, aligned and prepared in Brain Voyager for within - and between-group random effects analyses based on steps described by Goebel et al.[42].

### Preprocessing of functional data

Raw functional data (dicom format) was loaded and converted into Brain Voyager's internal "FMR" data format. Standard sequence of preprocessing steps including slice scan time correction, head motion correction, drift removal and spatial smoothing with Gaussian filter (FWHM = 5 mm) were conducted for each paradigm data set of each subject.

### Preprocessing of anatomical data

The anatomical data (dicom format) of each subject was loaded and converted into Brain Voyager's internal "VMR" data format. Intensity inhomogeneities correction was applied and the data was then resampled to 1-mm resolution, and transformed into AC-PC and Talairach standard space. The three spatial transformations were combined and applied backward in one step to avoid quality loss due to successive data sampling. The two affine transformations, iso-voxel scaling and AC-PC transformation, were concatenated to form a single 4 × 4 transformation matrix. For each voxel coordinates in the target (Talairach) space a piece affine "Un-Talairah" step was performed, followed by application of the inverted spatial transformation matrix. The computed coordinates were used to sample the data points in the original 3-D space using sinc interpolation.

### Brain segmentation

For 3-D visualization, the brain was segmented from surrounding head tissue using an automatic "brain peeling" tool. The tool analyzes the local intensity histogram in small volumes (20 × 20 × 20 voxels) to define thresholds for an adaptive region-growing technique. This step resulted in the automatic labeling of voxels containing the white and gray matter of the brain, but also other high-intensity head tissue. The next step consisted of a sequence of morphological erosions to remove tissue at the border of the segmented data. By "shrinking" the segmented data, this step separated subparts, which were connected by relatively thin "bridges" with each other. By determining the largest connected component after the erosion step, the brain was separated from other head tissue. Finally, the sequence of erosions was reversed but restricted to voxels in the neighborhood of the largest connected component.

### Cortex segmentation

In order to perform a cortex-based data analysis, the gray/white matter boundary was segmented using largely automatic segmentation routines [43]. Following the correction of inhomogeneities of signal intensity across space as described above, the white/gray matter border was segmented with a region-growing method using an analysis of intensity histograms. Morphological operations were used to smooth the borders of the segmented data and to separate the left from the right hemisphere. Each segmented hemisphere was finally submitted to a "bridge removal" algorithm, which ensures the creation of topologically correct mesh representations [43]. The borders of the two resulting segmented subvolumes were tessellated to produce a surface reconstruction of the left and right hemisphere. For better visualization of the areas of activities including those in the sulcus, the resulting meshes were transformed into inflated cortical representations by performing repeated small morphing steps until the central sulcus are visible. The inflated cortical meshes were used as the reference meshes for functional data (maps and time courses) projection. For subsequent cortex-based analysis, the inflated cortical meshes were used to sample the functional data at each vertex (node), resulting in a mesh time course ("MTC") dataset for each run of each subject.

### Normalization of functional data

To transform the functional data into Talairach space, the functional time series data of each subject was first coregistered with the subject's 3-D anatomical dataset, followed by the application of the same transformation steps as performed for the 3-D anatomical dataset (see above). This step results in normalized 4-D volume time course ("VTC") data. In order to avoid quality loss due to successive data sampling, normalization was performed in a single step combining a functional-anatomical affine transformation matrix, a rigid-body AC-PC transformation matrix, and a piecewise affine Talairach grid scaling step. As described for the anatomical normalization procedure, these steps were performed backward, starting with a voxel in Talairach space and sampling the corresponding data in the original functional space. In the context of the functional-anatomical alignment, some manual adjustment was necessary to reduce as much as possible the geometrical distortions of the echo-planar images, which exhibited linear scaling in the phase-encoding direction. The necessary scaling adjustment was done interactively using appropriate transformation and visualization tools of Brain Voyager QX.

### GLM analysis

For each run of each subject's block, a protocol file (PRT) was derived representing the onset and duration of the events for the different stimulation conditions. In order to account for hemodynamic delay and dispersion, each of the predictors was derived by convolution of an appropriate box-car waveform with a double-gamma hemodynamic response function [44] to extracted brain regions with both positively and negatively correlated blood oxygen level dependent (BOLD) responses. Within group random effect analysis was conducted for each paradigm and areas of activation (positively correlated BOLD) and deactivation (negatively correlated BOLD) were recorded. Between-group random effect analyses were also performed between MI-EA and OI-EA paradigms and a second level 2-factor ANOVA (EA and intensity) was also performed to assess treatment and intensity interaction effect at regions of interest related to HP stimulation.

## Competing interests

The authors declare that they have no competing interests.

## Authors' contributions

AL carried out the experiment, supervised the data analysis and prepared the manuscript. SS carried the data analysis, and graphic and manuscript preparation. AT and JR assisted in conducting the experiment and data analysis. All authors read and approved the final manuscript.
